# A Case of Acute Purulent Streptococcus pneumoniae Pericarditis Causing Tamponade and Cardiac Arrest in a COVID-19-Infected Patient

**DOI:** 10.7759/cureus.39467

**Published:** 2023-05-25

**Authors:** Omar M Masarweh, Parag Vyas, Thomas M Knapp, Ubaldo Gonzalez-Morales, Ali Ammar

**Affiliations:** 1 Internal Medicine, University of Central Florida, Kissimmee, USA; 2 Medicine, University of Central Florida College of Medicine, Orlando, USA; 3 Internal Medicine, University of Central Florida, Orlando, USA; 4 Cardiology, Orlando Health, Orlando, USA

**Keywords:** streptococcus pneumoniae, in hospital cardiac arrest, covid 19, pericardial effusion. cardiac tamponade, transthoracic echocardiogram, pericardiocentesis, purulent pericarditis

## Abstract

Purulent pericarditis due to *Streptococcus pneumoniae* *(S. pneumoniae)* has been increasingly rare since the advent of antibiotics; however, it still carries a high mortality rate, especially in the setting of tamponade. Bedside transthoracic echocardiogram (TTE) is a useful, cheap, and underutilized tool that can aid in the diagnosis, treatment, and further management of patients presenting to the emergency department with chest pain, as well as during cardiac resuscitation. In this report, we present a case of an acute purulent *S. pneumoniae* pericarditis of an unknown primary source in a patient coinfected with coronavirus disease 2019 (COVID-19) pneumonia, resulting in cardiac tamponade and cardiac arrest that resolved with the aid of bedside echocardiography-guided pericardiocentesis. We attempt to highlight the importance of clinicians using echocardiography to aid in their clinical decision-making, demonstrating it as a fast and effective tool capable of providing instantaneous feedback.

## Introduction

*Streptococcus pneumoniae* *(S. pneumoniae)* is a common cause of bacterial pneumonia in adults. Since the emergence of coronavirus disease 2019 (COVID-19), the number of hospitalizations due to pneumonia has risen in the last couple of years. *S. pneumoniae* can cause localized bacterial pneumonia resulting in complications such as empyema, pericarditis, bacteremia, and abscess formation [1]. Translocation of *S. pneumoniae* bacteria into the pericardium is a proposed mechanism of *S. pneumoniae* pericarditis, as well as hematogenous spread from a distal infection such as cellulitis. Acute purulent pericarditis secondary to *S. pneumoniae* has been decreasing in incidence since the advent of antibiotics and is rarely seen now. While the mortality rates were as high as 100% earlier, emergent identification and treatment have reduced the overall mortality to 40% [2]. Since 2019, there have been over 700 million COVID-19 cases worldwide, with over six million deaths estimated [3]. This new viral infection has been associated with the emergence of multiple coinfections and complications, such as superimposed infections. Their long-term side effects are still being identified. In this report, we present a COVID-19 patient with *S. pneumoniae* coinfection with no identifiable focus who developed acute purulent pericarditis that resulted in cardiac tamponade and cardiac arrest. The timely use of a bedside echocardiogram and subsequent immediate pericardial drainage was instrumental in the care and eventual improvement of this patient.

## Case presentation

**Figure 1:**
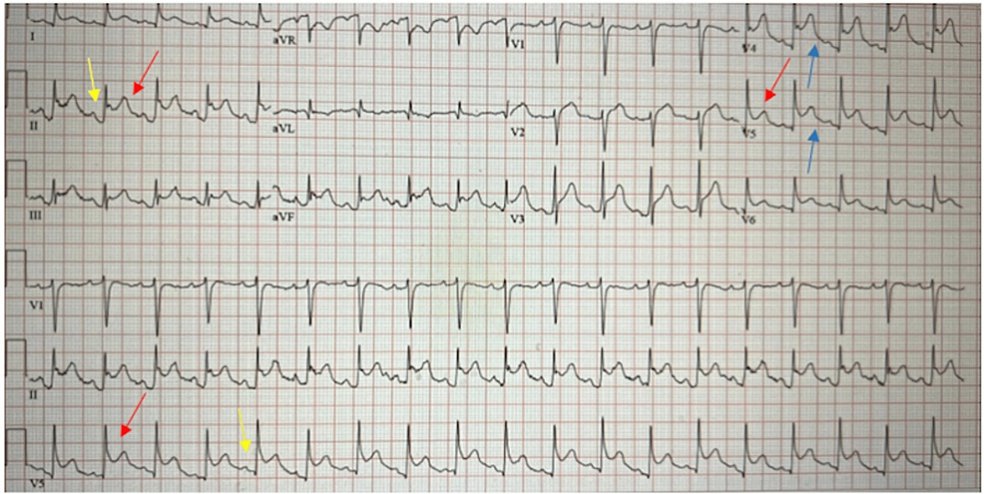
Electrocardiogram on initial presentation The image shows diffuse ST elevations (red arrow), PR depressions (yellow arrow), and Spodick's sign (blue arrow), all indicative of pericarditis

A 61-year-old male presented to the emergency department with two weeks of left-sided pleuritic chest pain and worsening shortness of breath with minimal exertion. On initial evaluation, he appeared comfortable lying in bed, and auscultation of his chest revealed mildly decreased heart sounds and fine rhonchi in bilateral lung bases. His vitals on presentation were as follows: a temperature of 99.6 °F, blood pressure of 131/85 mmHg, and heart rate of 117 beats per minute with 95% O_2_ saturation on room air. Electrocardiogram showed diffuse ST elevations in leads 1, 2, 3, AVF, and V2 to V6, ST depression in AVR, and PR depressions in 1,2,3, AVF, and V3 to V6 (Figure 1). Initial laboratory investigations revealed a leukocytosis of 13,000 cells/mm^3^ and a serum creatinine of 2.0 mEq/L with previously normal creatinine levels. All other initial labs were negative including two high-sensitivity troponins.

**Figure 2:**
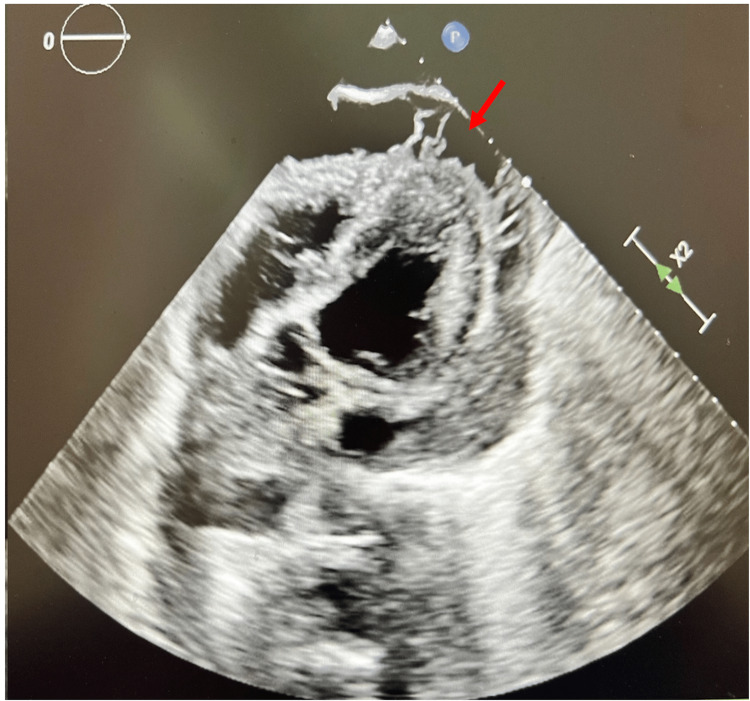
Immediate post-code bedside transthoracic echocardiogram showing a pericardial effusion (red arrow)

Chest X-ray demonstrated mild bilateral pulmonary infiltrates and the patient tested positive for severe acute respiratory syndrome coronavirus 2 (SARS-CoV-2). A few hours after imaging, the patient went into cardiac arrest with pulseless electrical activity and achieved return of spontaneous circulation (ROSC) after three rounds of cardiopulmonary resuscitation and two doses of epinephrine. During the cardiac resuscitation attempts, a bedside transthoracic echocardiogram (TTE) revealed a large pericardial fluid collection (Figure 2). Emergent pericardiocentesis was performed, yielding 200 ml of yellow purulent fluid. Laboratory analysis of a sample yielded a decreased pH and glucose, and an elevated total protein, lactate dehydrogenase, and white blood cells with culture positive for *S. pneumoniae* (Table 1). After the removal of the pericardial fluid and stabilization, the patient was started on vancomycin, azithromycin, and cefepime until the cultures resulted. After the identification of the organism with sensitivities, he was switched to ceftriaxone while still an inpatient. Blood cultures remained negative. Initial treatment of intravenous aspirin 400 mg twice a day and colchicine was administered for acute pericarditis. Immediately post-code, he was given norepinephrine for hypotension and was successfully extubated and taken off ionotropic support the following day. The next day, the TTE showed an ejection fraction of less than 10% with mildly increased left ventricular wall thickness and reduced right ventricular systolic function, without any additional fluid in the pericardial space. The patient made a full recovery and was transitioned to cefdinir for four weeks as well as metoprolol tartrate. He had one follow-up appointment after this hospitalization and was found to be doing well after the completion of his antibiotics. A repeat echocardiogram six months later did not show any recurrence of the pericardial effusion, and the ejection fraction had improved to 45-50%.

**Table 1:** Pericardial fluid analysis *Abnormal

Variables	Value	Reference
Color	Yellow purulent*	Clear/straw-colored
pH	5.34*	6.8-7.5
Glucose (mg/dL)	<10*	80-110
Protein (g/dL)	4.6*	1-2
Lactate dehydrogenase (u/L)	3,347*	275-510
White blood cells (cells/mm^3^)	16,000*	<300

## Discussion

The incidence of *S. pneumoniae* in the United States has dwindled over the past few decades thanks to antibiotics. Yet, it remains the most common cause of community-acquired pneumonia [4]. Contiguous spread of the organism to the pericardium in the context of pneumonia infection as its source is the most common scenario; however, it is still exceedingly rare with an approximated incidence of 9% over the last two decades [4,5]. The majority of the cases of pericarditis are found in children, immunocompromised patients, or when a source of infection is identified, such as pneumonia, which represents 93% of cases [2]. Though the patient presented in this report experienced chest pain as a symptom, most cases of purulent pericarditis do not present with chest pain [5-7]. Therefore, other distinguishing aspects of the disease must be ascertained to reach a diagnosis. When *S. pneumoniae* is the cause of pericarditis, an inciting event such as pneumonia or empyema is usually present as the initial source. Typically, it arises from hematogenous spread, such as cellulitis causing bacteremia, or contiguous spread from an intrathoracic process, such as pneumonia [8]. In this case, the patient had pneumonia; however, the pneumonia was caused by COVID-19, and sputum cultures were negative for *S. pneumoniae*. Although sputum cultures failed to grow a specific bacteria, there is a considerable rate of false negative sputum cultures with bacterial pneumonia, and therefore the possibility of a bacterial superinfection on top of COVID-19 remains a possibility. Chest X-ray showed no evidence of a consolidation suggesting bacterial pneumonia. Swift diagnosis and treatment of *S. pneumoniae* pericarditis is imperative as it carries a mortality rate of approximately 30% [1].

TTE is an underused imaging modality in life-threatening cardiovascular hospitalizations, which can provide critical information on the presence and extent of pericardial effusion [9,10]. Findings supportive of a diagnosis of pericarditis include characteristic pleuritic chest pain, ST elevations and PR depressions on EKG, and a pericardial friction rub heard on auscultation [10,11]. According to the literature, two out of four of these criteria should be fulfilled; however, a majority of patients will not report characteristic chest pain (sharp, worse with leaning forward and with deep breaths, better with sitting up) or elicit a friction rub on auscultation [5,12-15]. In this case, relevant EKG changes and chest pain were present, and TTE was performed at the bedside after their cardiac arrest. Bedside TTE is as effective as formal transthoracic electrocardiography in detecting pericardial effusion. In a study by Balderston et al., there was a 77% agreement for reduced left ventricular function and pericardial effusion between formal TTE by trained sonographers and bedside TTE [16]. Comparable accuracy to that of formal sonography indicates that bedside TTE is a useful immediate tool to guide the management of patients with pericardial effusion [16]. In our patient, TTE enabled the clinicians to determine that the patient’s cardiac arrest was caused by a pericardial effusion culminating in cardiac tamponade.

Pericardiocentesis with echocardiogram guidance functions as both a diagnostic and therapeutic tool for cardiac tamponade. Common etiologies of cardiac tamponade requiring pericardiocentesis are idiopathic and malignancies. Although purulent pericarditis is rare, it has a high mortality rate if left untreated. Therefore, early pericardiocentesis is heavily indicated in patients suspected of having pericardial effusions, as a life-saving measure. Diagnostically, especially in cases of coinfection such as this, it provides information regarding which infectious agent, viral or bacterial, is responsible for the inflammation of the pericardium and pericardial effusions. The incidence of pericardial effusions in SARS-CoV-2 pneumonia patients is 7.3%, which can be a substantial number of patients during times of peak total cases and hospitalizations [17]. Distinguishing between bacterial and viral pericarditis in the case of tamponade guides clinical management and selection of antibiotics. A positive culture of *S. pneumoniae* guided the medical team to initiate a regimen of vancomycin, azithromycin, and cefepime before transitioning to cefdinir for four weeks in our patient.

There are a few cases of coinfection of *S. pneumoniae* and SARS-CoV-2 in the literature. Synergism between these bacterial and viral agents in an acute presentation of a patient is possible. In studies from the United Kingdom, coinfection resulted in a significant increase in mortality [18,19]. A potential mechanism of the interaction may be the alteration of IgA and cell-mediated immunity by *S. pneumoniae*, allowing an optimal colonization opportunity for SARS-CoV-2 [19]. Although coinfection has been reported, coinfection causing purulent pericarditis and tamponade has yet to be reported. This case is unique as there was no focus on the identified *S. pneumoniae* including no evidence of skin or soft tissue infection, pneumonia, or bacteremia. To our knowledge, only eight other similar cases have been reported so far [8]. Clinicians should be aware of the possibility of the opportunistic conditions created by *S. pneumoniae* in favor of this now prominent and potentially fatal viral agent and further research is needed to correctly optimize treatments.

## Conclusions

The global spread of SARS-CoV-2 since the pandemic's outbreak has enabled possible synergistic coinfections with other infectious agents. Diagnostic actions to determine the underlying causes of pneumonia, pericarditis, and other conditions remain critical in guiding patient management. Even though *S. pneumoniae* is rare, its fulminant presentation requires an urgent and measured response by healthcare professionals to avoid fatalities. Utilization of bedside TTE, pericardiocentesis, and antibiotic treatment should be immediately considered in these patients.
